# COHORT DIFFERENCES IN CHANGES IN LIFE SATISFACTION AMONG OLDER JAPANESE

**DOI:** 10.1093/geroni/igz038.2567

**Published:** 2019-11-08

**Authors:** Takeshi Nakagawa, Erika Kobayashi

**Affiliations:** 1 National Center for Geriatrics and Gerontology, Aichi, Japan; 2 Tokyo Metropolitan Institute of Gerontology, Tokyo, Japan

## Abstract

Life span research has been interested in how sociocultural contexts shape individual development and aging processes. Empirical studies have reported that later cohorts show higher levels of well-being. However, more recent studies indicate that cohort differences are not sustained in very late life. The present study examined whether cohort differences in well-being, as measured by life satisfaction, are observed in the young-old and old-old, and further explored potential determinants of cohort differences. Using longitudinal data from a nationally representative study of older Japanese, conducted from 1987—2002, we compared earlier- and later-born cohorts in the young-old (N = 874; age 60—65; year of birth: 1922—1927 and 1931—1936) and old-old (N = 1,022; age 70—80; year of birth: 1907—1917 and 1919—1929), respectively. To control for covariates, we used case-matched groups based on age and gender. Results revealed that later cohorts exhibited higher levels of life satisfaction in both age groups. In the young-old, life satisfaction declined across cohorts. In the old-old, life satisfaction remained stable among earlier cohorts but declined among later cohorts. Socioeconomic, social, and health factors at the individual level and methodological factors (i.e., number of observations) did not fully explain the cohort differences in both age groups. Our results suggest that historical increases in levels of well-being are observed in late life, but that these improvements do not hold in very late life. Future studies should consider potential societal factors behind observed cohort differences in well-being.

